# Coding-complete genomes of 18 SARS-CoV-2 Omicron JN.1, JN.1.4, and JN.1.11 sub-lineages in Bangladesh

**DOI:** 10.1128/mra.00135-24

**Published:** 2024-04-24

**Authors:** Anika Tahsin, Mahin Hasan, Saikt Rahman, Mohammad Jubair, Mokibul Hassan Afrad, Manjur Hossain Khan, Md Shaheen Alam, Mst Noorjahan Begum, Md Yeasir Karim, Sharmin Aktar Mukta, Mohammad Tanbir Habib, Ahmed Nawsher Alam, Emran Kabir Chowdhury, Md Rofiqur Rahman, Edward T. Ryan, Tahmina Shirin, Mustafizur Rahman, Firdausi Qadri

**Affiliations:** 1 Institute for Developing Science and Health initiatives, Dhaka, Bangladesh; 2 International Centre for Diarrhoeal Disease Research, Dhaka, Bangladesh; 3 Institute of Epidemiology, Disease Control, and Research, Dhaka, Bangladesh; 4 Department of Biochemistry and Molecular Biology, University of Dhaka, Dhaka, Bangladesh; 5 Division of Infectious Diseases, Massachusetts General Hospital, Boston, Massachusetts, USA; 6 Department of Medicine, Harvard Medical School, Boston, Massachusetts, USA; 7 Department of Immunology and Infectious Diseases, Harvard T.H. Chan School of Public Health, Boston, Massachusetts, USA; DOE Joint Genome Institute, Berkeley, California, USA

**Keywords:** SARS-CoV-2, JN.1 sub-lineages, coding-complete, ONT, variant surveillance

## Abstract

We report 18 coding-complete genome sequences of emerging SARS-CoV-2 Omicron sub-lineages JN.1, JN.1.4, and JN.1.11 from Bangladesh. Nasopharyngeal swab samples were obtained from individuals with COVID-19 symptoms between December 2023 and January 2024. Whole genome sequencing was performed following the ARTIC Network-based protocol using Oxford Nanopore Technology.

## ANNOUNCEMENT

Persistent transmission of SARS-CoV-2 (genus *Betacoronavirus*, family *Coronaviridae*), the virus responsible for COVID-19, facilitates emergence of new variants escalating public health concerns. A probable descendant of Omicron BA.2.86, JN.1, is non-recombinant in genetic structure and is currently surpassing the cases of its recombinant XBB lineages (1). The World Health Organization announced JN.1 as a Variant of Interest due to its rapid evolution, giving rise to sub-lineages such as JN.1.4 and JN.1.11 (2, 3).

Nasopharyngeal swab specimens were collected from individuals across the country with COVID-19 symptoms (Table 1) as routine SARS-CoV-2 diagnostic sampling for the Respiratory Virus Genomic Surveillance in Bangladesh [Institute of Epidemiology, Disease Control, and Research (IEDCR) protocol IEDCR 14,26.08.2021]. QIAamp viral RNA mini kit (Qiagen, Germany) was used for viral RNA extraction, followed by real-time reverse transcription polymerase chain reaction (rRT-PCR) using a nucleic acid diagnostic kit (Sansure Biotech, China) targeting novel coronavirus (2019-nCoV). From the routine surveillance, 18 positive samples with a cycle threshold (C_T_) value <27 were selected for whole genome sequencing according to the amplicon-based SARS-CoV-2 sequencing protocol established by ARTIC Network (4).

**TABLE 1:** Sampling and sequencing information of the identified SARS-CoV-2 Omicron JN.1 sub-lineages

Sequence ID	Date of sample collection (mm/dd/yyyy)	Location	Sex^*a*^	Age	Vaccination	Number of doses	GenBank_ID	GISAID ID	SRA ID	Lineage	No. of reads	Read depth	Median read length (bp)	Genome length	GC content (%)
SIRS_3231203017	12/22/2023	Kishoreganj	F	1 year	no	N/A	PP231959	EPI_ISL_18839342	SRR27797267	JN.1	175,510	3060.75	485	29,709	37.91
SIRS_2240113003	1/3/2024	Chattogram	M	48 years	yes	>2	PP231962	EPI_ISL_18839347	SRR27797253	JN.1	147,422	2537.17	473	29,709	37.89
SIRS_2240113004	1/3/2024	Chattogram	F	40 years	yes	>2	PP231965	EPI_ISL_18839348	SRR27797252	JN.1.4	136,389	2328.47	474	29,709	37.88
SIRS_2240103007	1/6/2024	Kishoreganj	M	37 years	yes	>2	PP231961	EPI_ISL_18839343	SRR27797257	JN.1	141,683	2431.25	473	29,709	37.91
SIRS_3240111007	1/6/2024	Chattogram	M	2 months 15 days	no	N/A	PP231968	EPI_ISL_18839352	SRR27797265	JN.1	64,018	1088.62	473	29,709	37.87
SIRS_2240111009	1/8/2024	Sylhet	F	5 years	no	N/A	PP231964	EPI_ISL_18839346	SRR27797254	JN.1	170,325	2947.39	476	29,709	37.87
SIRS_2240113010	1/8/2024	Chattogram	F	4 years 1 month	no	N/A	PP231967	EPI_ISL_18839349	SRR27797251	JN.1	105,880	1837.19	477	29,709	37.88
SIRS_3240113010	1/8/2024	Chattogram	F	1 month 8 days	no	N/A	PP231970	EPI_ISL_18839353	SRR27797264	JN.1	98,371	1698.62	475	29,709	37.88
SIRS_1240108008	1/9/2024	Cumilla	F	7 years	no	N/A	PP231960	EPI_ISL_18839344	SRR27797256	JN.1	117,614	2011.9	477	29,709	37.88
SIRS_2240114009	1/9/2024	Dinajpur	M	18 years	yes	2	PP231969	EPI_ISL_18839351	SRR27797266	JN.1	134,461	2286.05	470	29,709	37.88
SIRS_1240110006	1/10/2024	Jashore	F	38 years	yes	>2	PP231963	EPI_ISL_18839345	SRR27797255	JN.1.11	168,461	2887.35	472	29,709	37.84
SIRS_3240113013	1/10/2024	Chattogram	F	1 year 5 months	no	N/A	PP231971	EPI_ISL_18839354	SRR27797263	JN.1	128,548	2204.56	474	29,709	37.88
SIRS_2240113014	1/10/2024	Chattogram	F	45 years	yes	>2	PP231966	EPI_ISL_18839350	SRR27797250	JN.1	167,464	2862.94	475	29,709	37.89
SIRS_3240113015	1/11/2024	Chattogram	M	4 months 5 days	no	N/A	PP231973	EPI_ISL_18839355	SRR27797262	JN.1	132,742	2305.08	476	29,709	37.87
SIRS_2240111020	1/15/2024	Sylhet	F	1 year 3 months	no	N/A	PP231975	EPI_ISL_18839357	SRR27797260	JN.1	44,085	692.74	467	29,700	37.83
SIRS_3240113019	1/16/2024	Chattogram	M	3 months 15 days	no	N/A	PP231976	EPI_ISL_18839359	SRR27797258	JN.1	81,437	1375.79	470	29,709	37.88
SIRS_1240108019	1/20/2024	Cumilla	F	15 years	yes	2	PP231972	EPI_ISL_18839356	SRR27797261	JN.1	35,228	547.55	468	29,415	37.75
SIRS_3240113025	1/20/2024	Chattogram	M	8 months 26 days	no	N/A	PP231974	EPI_ISL_18839358	SRR27797259	JN.1	105,253	1806.47	473	29,709	37.88

^*a*^ F, female; M, male.

Briefly, viral cDNA synthesis using LunaScript RT SuperMix Kit (New England Biolabs, USA) was followed by sequencing library preparation via multiplex PCR with Q5 high-fidelity DNA polymerase (New England Biolabs, USA) using ARTIC v4.1 primer panels. Amplicons were treated with NEBNext Ultra II end repair/dA-tailing module (New England Biolabs, USA). Following native barcode (EXP-NBD104 and EXP-NBD114) ligation (Oxford Nanopore Technologies, UK) with blunt/TA DNA ligase (New England Biolabs, USA), amplicons were pooled into a single reaction tube. Upon adapter ligation with NEBNext Quick T4 DNA ligase (New England Biolabs, USA), the final DNA library was loaded onto a FLO-MIN106D flow cell (R9.4.1). Sequencing was performed on a MinION Mk1C sequencing device with the default quality score filter (Q-score = 8) for at least 6 hours. Demultiplexing and base calling of raw reads were performed using the Fast base-calling model on MinKNOW v22.12.5 (bionic). ARTIC guppyplex code script was employed for assembling reads thus processed. Consensus sequences in fasta format were constructed on ARTIC EPI2ME desktop agent v3.7.3 using Medaka v1.4 (https://artic.network/ncov-2019/ncov2019-bioinformatics-sop.html).

In total, 2,154,891 reads were produced with a range of 35,228 to 175,510 reads per sample. For each sample, nearly an end-to-end genome was achieved. Information on sampling and sequencing are presented in Table 1. Lineages of the viruses sequenced were assigned according to the proposed nomenclature on the Pangolin lineage assignment software v4.3.1. (FastQ quality control > ARTIC > NextClade v2023.06.10-1862208) (https://github.com/cov-lineages/pangolin). All bioinformatics tools were run on parameters set by default.

In comparison with the reference Wuhan Hu-1 SARS-CoV-2 genome (GenBank accession NC_045512.2), JN.1 evolved with an additional spike-protein amino acid substitution (L455S) to its parent BA.2.86 and is currently responsible for the highest incidence of SARS-CoV-2 infection worldwide (5). SARS-CoV-2 Omicron BA.2.86 is characterized by significant evolutionary drift with more than 30 spike-glycoprotein mutations compared with its ancestor Omicron BA.2, denoting a high potential for immune evasion (6, 7). JN.1.4 and JN.1.11 are JN.1 descendants with a distinct amino acid variation in ORF1a-protein (T1701I) and spike-protein (V1104L), respectively. In Bangladesh, circulation of these variants in such a brief timeline might indicate their introduction through independent sources. Accordingly, genomic surveillance must be continued to monitor emerging SARS-CoV-2 strains for effective planning and implementation of public health policies.

## Data Availability

The genome data generated in this study are available through the GISAID accession numbers, the NCBI GenBank accession numbers, and the SRA accession numbers listed in Table 1.
